# Multidomain chimeric enzymes as a promising alternative for biocatalysts improvement: a minireview

**DOI:** 10.1007/s11033-024-09332-9

**Published:** 2024-03-11

**Authors:** Flor de María García-Paz, Sandra Del Moral, Sandra Morales-Arrieta, Marcela Ayala, Luis Gerardo Treviño-Quintanilla, Clarita Olvera-Carranza

**Affiliations:** 1 Departamento de Ingeniería Celular y Biocatálisis, Instituto de Biotecnología, Universidad Nacional Autónoma de México, Av. Universidad 2001 Col. Chamilpa CP 62210, Cuernavaca, Morelos México; 2 Investigador por México-CONAHCyT, Unidad de Investigación y Desarrollo en Alimentos, Tecnológico Nacional de México, Campus Veracruz. MA de Quevedo 2779, Col. Formando Hogar, CP 91960 Veracruz, Veracruz México; 3 Departamento de Biotecnología, Universidad Politécnica del Estado de Morelos, Boulevard Cuauhnáhuac No. 566 Col. Lomas del Texcal CP 62550, Jiutepec, Morelos México

**Keywords:** Chimeric enzymes, Fusion enzymes, Enzyme activity, Enzyme stability, Multidomain enzymes

## Abstract

Searching for new and better biocatalysts is an area of study in constant development. In nature, mechanisms generally occurring in evolution, such as genetic duplication, recombination, and natural selection processes, produce various enzymes with different architectures and properties. The recombination of genes that code proteins produces multidomain chimeric enzymes that contain two or more domains that sometimes enhance their catalytic properties. Protein engineering has mimicked this process to enhance catalytic activity and the global stability of enzymes, searching for new and better biocatalysts. Here, we present and discuss examples from both natural and synthetic multidomain chimeric enzymes and how additional domains heighten their stability and catalytic activity. Moreover, we also describe progress in developing new biocatalysts using synthetic fusion enzymes and revise some methodological strategies to improve their biological fitness.

## Introduction

The enzymatic catalysts generate hundreds of compounds of industrial interest; thus, the enhancement of biocatalysts is an area of study in constant development to increase yield in the production process. Biocatalysts with better properties have been obtained through several strategies, such as chemical modification, rational design, and directed evolution, that modify enzyme properties. Researchers aim to get robust biocatalysts displaying essential properties for practical applications such as storage and operational stability, reusability, high catalytic efficiency, and specificity, among others [1].

Genetic recombination, which has occurred in nature over millions of years of evolution, has generated variability of proteins that mix different regions or take regions from other proteins and incorporate them into their sequences [2]. The establishment of multidomain chimeric enzymes results from combining two or more structural or functional domains from different proteins, understanding a protein domain as a structural polypeptide unit having a specific and independent folding and function. The wide distribution of multidomain proteins in nature reflects the success of multidomain combinations. For example, in the kingdoms of archaea and bacteria, 40% of proteins have more than one domain within this group; 20% contain two domains, and another 20% are multidomain proteins with three or more domains. Similarly, in the eukaryotic kingdom, 65% of proteins contain more than one domain: 20% have two domains, while 45% have three or more domains. This large percentage of multidomain proteins in higher organisms suggests that one of the mechanisms for diversification functions could be incorporating domains into these proteins [3].

The diversity of reactions catalyzed by multidomain enzymes and the exciting properties conferred by their additional domains have inspired molecular engineers to turn to protein engineering and use those domains as building blocks to create chimeric enzymes in search of ideal biocatalysts. This review shows examples of chimeric enzymes in nature, focusing on how additional domain incorporation modulates their enzyme stability, catalytic activity, and, in some cases, enzyme specificity. It also describes approaches to constructing synthetic chimeric enzymes to improve their properties. Finally, we describe the progress in developing new biocatalysts that generate bifunctional chimeric enzymes and some methodological strategies for constructing and improving their biological fitness.

## Multidomain enzymes in nature

**Fig. 1:**
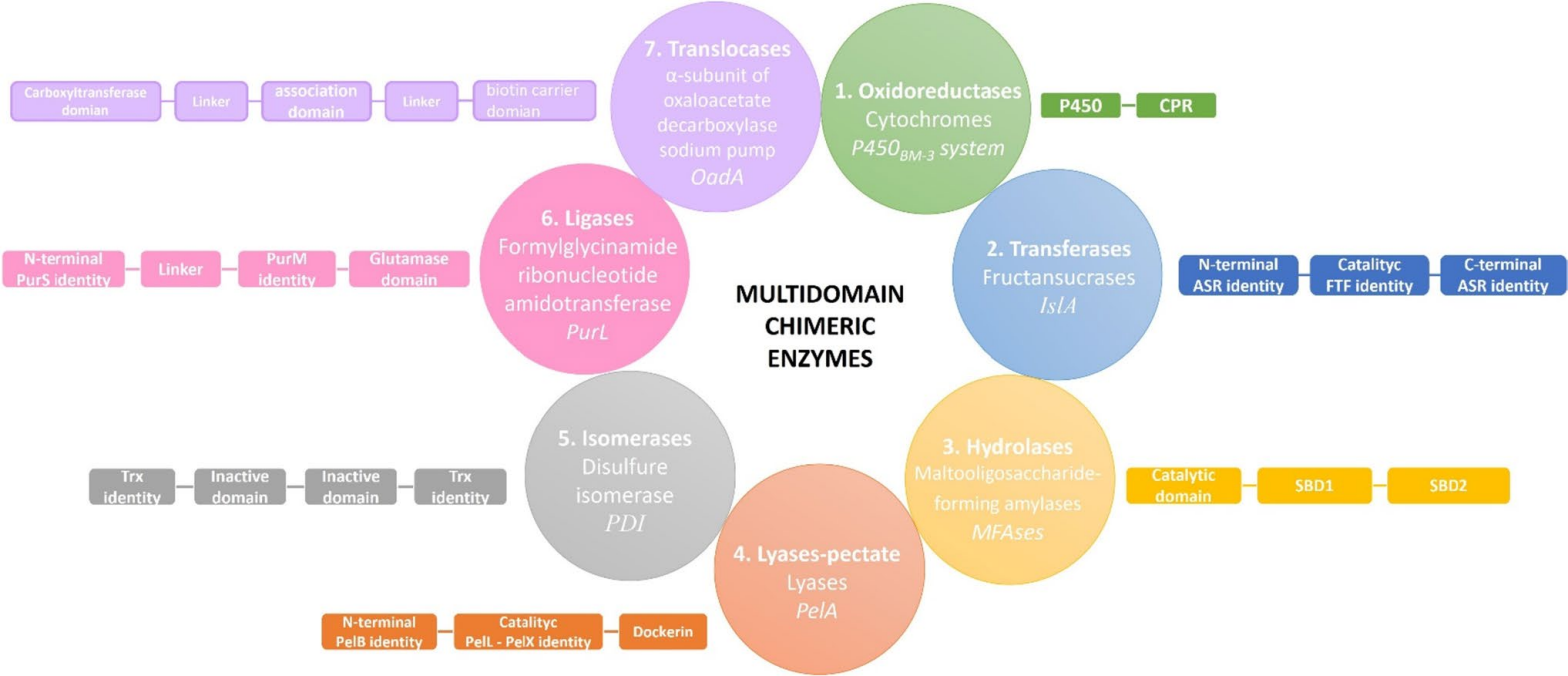
Multidomain chimeric enzymes in nature. Examples of multidomain enzymes in nature belonging to the seven enzyme classes

There are countless examples of multidomain enzymes belonging to each of the seven enzyme classes in nature: oxidoreductases, transferases, hydrolases, lyases, isomerases, ligases, and translocases. We will now describe and discuss an illustrative example of each class of multidomain enzymes in nature (Fig. 1).

*Bacillus megaterium* cytochrome P450_BM − 3_ (EC 1.14.-) is a representative enzyme of a multidomain oxidoreductase [4] containing two domains, Fig. 1. The classical P450 reaction introduces an oxygen atom (from molecular oxygen) to produce hydroxylation at an inactivated carbon on a molecule. The electrons for catalysis derive (usually) from NAD(P)H and are delivered from one or more redox partner enzymes, as shown below:

1 $$RH{\text{ }} + {\text{ }}O_{2} + {\text{ }}H^{ + } + {\text{ }}NADPH{\text{ }} \to {\text{ }}ROH{\text{ }} + {\text{ }}H_{2} O{\text{ }} + {\text{ }}NADP^{ + }$$

In the most frequently characterized P450 redox systems, the electrons are transferred through NADPH-dependent cytochrome P450 reductase (CPR). The flavocytochrome P450_BM − 3_ system (CYP102A1) from *B. megaterium* is a fusion between two domains from two enzymes with complementary functions: a P450 and a rat CPR domain. This multidomain architecture allows the enzyme to catalyze the entire monooxygenase reaction of a substrate with the addition of only NADPH and O_2_. The natural domain fusion arrangement of the cytochrome P450_BM − 3_ produced a very fast enzyme that oxygenates arachidonic acid at ∼17,000 min^−1^ with a more efficient inter-cofactor electron transfer and higher catalytic activity than other P450 monooxygenase enzymes [5]. Many researchers have reconstituted functional P450 enzymes by fusion with different reductase proteins, some with improved properties; Sadeghi et al., 2013, have summarized these studies [6].

There is a wide diversity of natural multidomain transferases; some examples of these natural multidomain enzymes are the fructansucrases from lactic acid bacteria. These enzymes catalyze the transfer of the fructosyl unit from sucrose to either a growing fructan polymer chain (transglycosylase activity) or to water (hydrolytic activity). Among this enzyme group, there is the fructansucrase IslA (EC 2.4.1.9), a glycosyl hydrolase synthesized by *Leuconostoc citreum* CW28, able to produce inulin, a fructose polymer joined by β(2 − 1) linkages. This enzyme harbors three domains: the N-terminal domain, which shows identity (40%) with the alternansucrase ASR from *L. mesenteroides* NRRL B-1355; the catalytic domain is similar to single domain fructansucrases from several microorganisms; and the C-terminal, which shows identity (80%) with the C-terminal domain of ASR. The C-terminal domain consists of four related but non-identical tandem repeats of 20 to 30 amino acids, defined by their sequences and capable of binding polysaccharides [7]. IslA was the first natural chimeric glycosyltransferase in nature reported to have fructansucrase activity. Characterization of IslA truncated versions demonstrated that domain acquisition renders fructansucrases more stable and switches the reaction specificity, favoring the transglycosylase reaction over the hydrolytic reaction [8]. Combining domains seems to be a typical process in *Leuconostoc* fructansucrases since three other natural chimeric levansucrases have been reported: LevS in B512F strain as well as LevC and LevL in ATCC 8293 strain from *L. mesenteroides*. All these fructansucrases maintain a similar multidomain architecture to IslA; however, N- and C-terminal domains have identity with the glucansucrase DsrS from *L. mesenteroides* B512F. These additional domains also participate in the stability and reaction specificity of the enzyme, similar to IslA [9–11].

Regarding multidomain hydrolases, there is an extensive repertoire of enzymes; examples of these are maltooligosaccharide-forming amylases (MFAses) (EC 3.2.1.8, Fig. 1), which belong to glycosyl hydrolase family 13 and can hydrolyze starch into maltooligosaccharides, carbohydrates compounds of α-d-glucopyranosyl units linked by α-1,4 glycosidic linkages, usually with a degree polymerization of 2–10. MFAs commonly have a multidomain architecture because they contain starch-binding domains (SBDs). SBDs are structurally independent protein noncatalytic modules but generally enclose substrate binding sites to improve enzymatic performance. The function of SBDs appended to amylases for binding raw starch have been demonstrated through truncation, imaging, and molecular dynamics (MD) simulations [12]. An interesting case is maltooligosaccharide-forming amylase from *Saccharophagus degradans* (SdMFA), which contains a noncatalytic SBD that belongs to the carbohydrate-binding module family 20 and enables modulation of the product specificity. SdMFA exhibited a higher level of exo-action and greater product specificity when reacting with amylopectin than with amylose. Based on analysis of the product profile of truncated versions lacking C-terminal and fusion proteins of this region with MFA from *Bacillus megaterium*, the authors demonstrated that SBD contained in the C-terminal region of the SdMFA is responsible for the production of mainly GP 5 oligosaccharides. These data, plus molecular dynamics simulation, led the author to suggest that SBD could promote the recognition of nonreducing ends of substrates and delivery of the substrate chain to a groove end toward the active site in the catalytic domain [13].

PelA pectate lyase (EC 4.2.2.2) is a multidomain enzyme belonging to the lyase class. This enzyme is made up of an N-terminal domain partially homologous to a putative cellulose-binding domain present at the C-terminus of *Erwinia chrysanthemi* pectate lyase PelB, a catalytic domain homologous to *E. chrysanthemi* pectate lyases PelL and PelX, and a duplicated sequence at the C-terminus that is highly conserved in the enzyme subunits of the cellulosome of *C. cellulovorans*. This enzyme can cleave polygalacturonic acid to digalacturonic acid (G2) and trigalacturonic acid (G3) but cannot act on G2 and G3. Cleavage patterns using substrates of different lengths suggest that PelA is an endo-type enzyme, while its PelX counterpart is an exopolygalacturonate lyase. Therefore, the properties of PelA differ from those of PelL and PelX in the specific activity, substrates, and synthesized products, even though these enzymes belong to the same family of pectate lyases [14]. These differences are probably related to its multidomain structure.

Martinez et al., 2014, analyzed the architectures of 96 isomerases, finding that one-third include more than one domain, and a large majority contain two or three domains [15]. An example of multidomain isomerases is disulfide isomerase (PDI, Fig. 1) (EC 5.3.4.1), an essential folding catalyst and chaperone of the endoplasmic reticulum. This protein introduces disulfide bonds into proteins (oxidase activity) and catalyzes the rearrangement of incorrect disulfide bonds (isomerase activity). The PDI structure contains four domains. The first and fourth domains are homologous to thioredoxin, and both have a respective active site. The second and third are noncatalytic domains similar in sequence [16]. Based on functional studies of linear combinations of PDI domains, Darby et al. (1998) showed that all protein domains of PDI are required for maximum catalytic efficiency. This suggests that PDI has developed its multidomain structure as an adaptation that allows it to catalyze transformations involving unfavorable conformational changes more efficiently [17].

We select PurL (EC 6.3.5.3) to illustrate multidomain ligases. In Gram-positive bacteria and archaea, PurL is a member of the formylglycinamide ribonucleotide amidotransferase (FGAR-AT) complex constituted by three proteins (PurS, PurL, and PurQ). This complex catalyzes the ATP-dependent conversion of formylglycinamide ribonucleotide (FGAR) and glutamine to formylglycinamide ribonucleotide (FGAM), ADP, P_i_, and glutamate in the fourth step of the purine biosynthetic pathway. The structure of PurL reveals four domains: An N-terminal domain structurally homologous to a PurS dimer, a linker region, a FGAM synthetase domain homologous to the PurM dimer of aminoimidazole ribonucleotide synthetase, and a triad glutaminase domain. These domains are intricately linked by interdomain interactions and peptide connectors [18], supporting the hypothesis that all domains are indispensable for correct folding and, thus, to the activity of PurL.

Finally, we use microbial oxaloacetate decarboxylase sodium pump (OAD) to exemplify multidomain translocases (EC 7.2.4.2). This enzyme is present in bacteria and archaea and maintains the sodium gradient, anaerobic citrate fermentation, and pathogenesis [19]. OAD is a membrane-bound multiprotein complex composed of three subunits (α, β, and γ) that catalyze the transfer of the carboxyl group from position 4 of oxaloacetate to the biotin prosthetic group. The carboxy biotin formed is transferred from the carboxyltransferase catalytic site of the α subunit to the decarboxylase site on the transmembrane β subunit, where decarboxylation takes place, liberating the biotin group. This last reaction is Na^+^-dependent, where a proton is consumed, and two sodium ions are translocated from the cytoplasm into the periplasm [20]. The α-subunit (OadA) is a cytoplasmatic protein with three domains connected by a flexible linker. The N-terminal domain harbors the carboxyltransferase catalytic site; the C-terminal domain includes the biotin-binding residues; and the third is denominated association domain. The association domain of the β subunit binds tightly to the C-terminal domain of the γ-subunit, taking a critical role in the assembly and stability of the oxaloacetate decarboxylase complex [21].

**Fig. 2:**
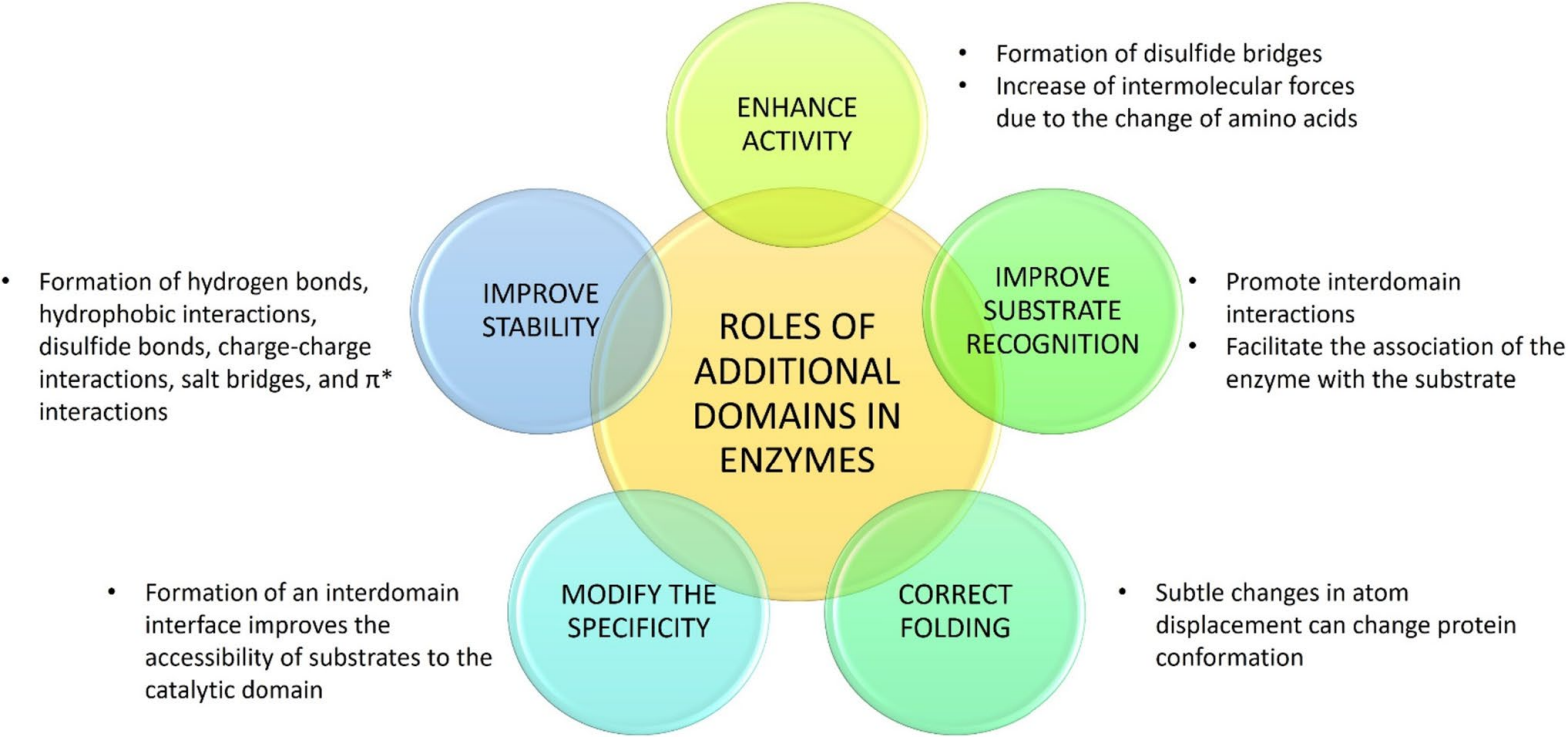
Functional roles of additional domains in multidomain enzymes

All these representative examples show that domain acquisition has modified enzyme properties such as substrate binding affinity, catalytic activity, and even enzyme specificity. Also, these domains can favor the correct folding and stability of the enzyme, with a consequent effect on the catalysis. Based on the importance of the additional domains of multidomain chimeric enzymes, we can infer that domain acquisition is an adaptive evolutionary process to expand and improve enzyme properties (Fig. 2). Therefore, it is possible to synthetically design and generate enzymes with higher activity and stability through a domain rearrangement process to produce better biocatalysts.

## Construction of chimeric enzymes: improving stability

Enzymes in the industry require that they be stable under process conditions such as high temperatures, extreme pH, and the presence of salts, surfactants, and solvents [22]. One of the biochemical characteristics favored in multidomain proteins is thermostability, which is achieved by stabilizing unstable regions by changing amino acids, placing disulfide bridges, or increasing intermolecular forces. However, some authors have reported the exchange of domains between enzymes to improve their stability, as shown in Table 1. For example, the N-terminal domain of xylanases (EC 3.2.1.8) has been reported as essential for enzyme stability. To improve the thermostability of xylanases, several research groups have performed a partial or total replacement of the N-terminal domain of mesophilic xylanases with a thermophilic counterpart, forming enzymes more thermostable up to 10 °C or more than their parental enzymes [23, 24].

**Table 1:** Synthetic chimeric enzymes with improved physicochemical properties over native enzymes

Enzyme	EC number	Organism	Domain Added	Domain origin	Improved Property	Refs.
Laccase	EC 1.10.3.2	*Pleurotus ostreatus*	Complete class I hydrophobin Vmh2	*Pleurotus ostreatus*	• Increased immobilization yield	[25]
Glicosylhidroolase	EC 3.2.1	*Thermotoga maritima*	Dockerin domain	*Piromyces finnis*	• Thermostability	[26]
Glucoamylase GATE	EC 3.2.1.3	*Talaromyces emersonii* Ld418 TE	Starch-Binding Domain of glucoamylase GAA1	*Apergillus niger* Ld418A1	• Enzyme activity • pH stability	[27]
Xylanase	EC 3.2.1.8	*Thermobacillus xylanilyticus*	N-terminal of GH11 xylanase	*Neocallimastix patriciarum*	• Wider substrate specificity	[28]
Xylanase A AnxA	EC 3.2.1.8	*Aspergillus niger*	N-terminal of xylanase A TfxA	*Thermomonospora fusca*	• Thermostability • Catalytic activity	[23]
β-xylanase	EC 3.2.1.8	*B. subtilis*	A complete β-xylosidase	*B. subtilis*	• Substrate cleavage rate • Optimum temperature • Thermostability	[29]
Cyanide dehydratase	EC 4.2.1.66	*B. pumilus*	C-terminal of cyanide dehydratase	*P. stutzeri*	• Thermostability • Optimum pH • Operational pH range	[30]
Nitrile hydratase	EC 4.2.1.84	*P. putida* NRRL-18,668	N-terminal of nitrile hydratase C-terminal of nitrile hydratase	*Comamonas testeroni* 5-MGAM-4D *P. thermofila* JCM3095	• Enhancement in thermostability • More tolerant to high-concentration product • Increase in activity	[31]

*CMB* Carbohydrate binding module *GH* Glycosyl hydrolase

The improvement in the stability can be reflected in more than one characteristic, as in the case of cyanide dehydratase (EC 4.2.1.66) from *Bacillus pumilus*, whose thermostability and pH tolerance were improved when the C-terminal domain was replaced by its homologous C-terminal domain (56 aa) of the thermostable *P. stutzeri* cyanide dehydratase. The half-life of the chimeric enzyme was increased 17-fold, and the optimum pH was 8–9. At this pH, wild-type cyanide dehydratase showed no activity; however, the chimeric enzyme showed an enhanced affinity for the substrate. This study suggests that oligomerization stimulated by the C-terminal domain was responsible for the longer half-life of the chimeric enzyme [30].

Proteins are stabilized by intermolecular forces such as hydrogen bonds, hydrophobic interactions, disulfide bonds, charge-charge interactions, salt bridges, and π* interactions. Although hydrogen bonds are the main force involved in protein structure formation, the hydrophobic patches are energetically the most stable. Some authors mention that the interior of a protein is very compact; therefore, its molecules are twice as close as in a water drop. Therefore, subtle changes in atom displacement can change protein conformation, increasing or decreasing its stability [32]. As seen in Table 1, chimerization can modify the pH and temperature stability at which enzymes remain stable. However, few studies used this strategy to improve enzyme stability in salts, ionic surfactant liquids, and organic or eutectic solvents. Due to thermal and pH stability determinants being similar to those involved in enzyme stabilization in salts, surfactants, or solvents, we consider this an opportunity for protein engineering to explore in the future.

## Construction of chimeric enzymes: improving activity

An optimum biocatalyst must have a high turnover number (k_cat_) or the maximum specificity constant (k_cat_/Km) in an efficient bioconversion reaction. Some strategies used to improve enzymatic activity during the last years are immobilization, organic solvents, and directed evolution. However, the design of chimeric enzymes is an exciting alternative to generate more efficient biocatalysts. Chimeric enzymes can be designed to synthesize new products by combining two or more catalytic features in a single molecule. These combinations sometimes convey an increase in catalytic activity and improve the effectiveness of these biocatalysts in developing bioprocess. Below are some examples to describe activity improvement by constructing chimeric enzymes.

Branchini et al. (2014) increased the bioluminescence properties of luciferase by constructing a chimeric enzyme (PpyLit) that contains the N-terminal domain of *Photinus pyralis* luciferase (EC 1.13.12.7) linked to the C-terminal domain of *Luciola italica* luciferase. PpyLit chimera exhibited 1.8-fold enhanced flash-height specific activity and a 2.9-fold improved catalytic efficiency (kcat/Km) compared to the *P. pyralis* enzyme. The enzyme conformation, where the N- and C-terminal domains interact, provided a favorable environment for an electronically excited state of oxyluciferin. Suggesting that the interactions between these domains generated an auxiliary microenvironment for the reaction mechanism [33].

Interdomain interactions also promote access of substrate molecules to the active site by increasing enzymatic activity. In 2012, the construction of three chimeric levansucrases from SacB, a single domain levansucrase produced by *B. subtilis*, was reported. These chimeric enzymes were formed by adding the transition region or entire C-terminal domain of the inulosucrase IslA (EC 2.4.1.9) from *L. citreum* CW28, the levansucrase LevC (EC 2.4.1.10) from *L. mesenteroides* ATCC 8293, and the glucansucrase DsrP (EC 2.4.1.5) from *L. mesenteroides* IBT-PQ. Adding the C-terminal domain of IslA and LevC increased transglycosylase activity by up to 90% compared to the wild-type SacB enzyme, similar to the transglycosylation activity of IslA. The authors suggest that the additional domains favor a fit in the catalytic domain that increases the affinity for the acceptor molecule. The addition of the C-terminal domain of DsrP did not affect the properties of SacB, indicating that there are molecular determinants that favor these changes. The stability of these chimeric enzymes was not affected, as it remained the same as that of SacB [34].

**Fig. 3:**
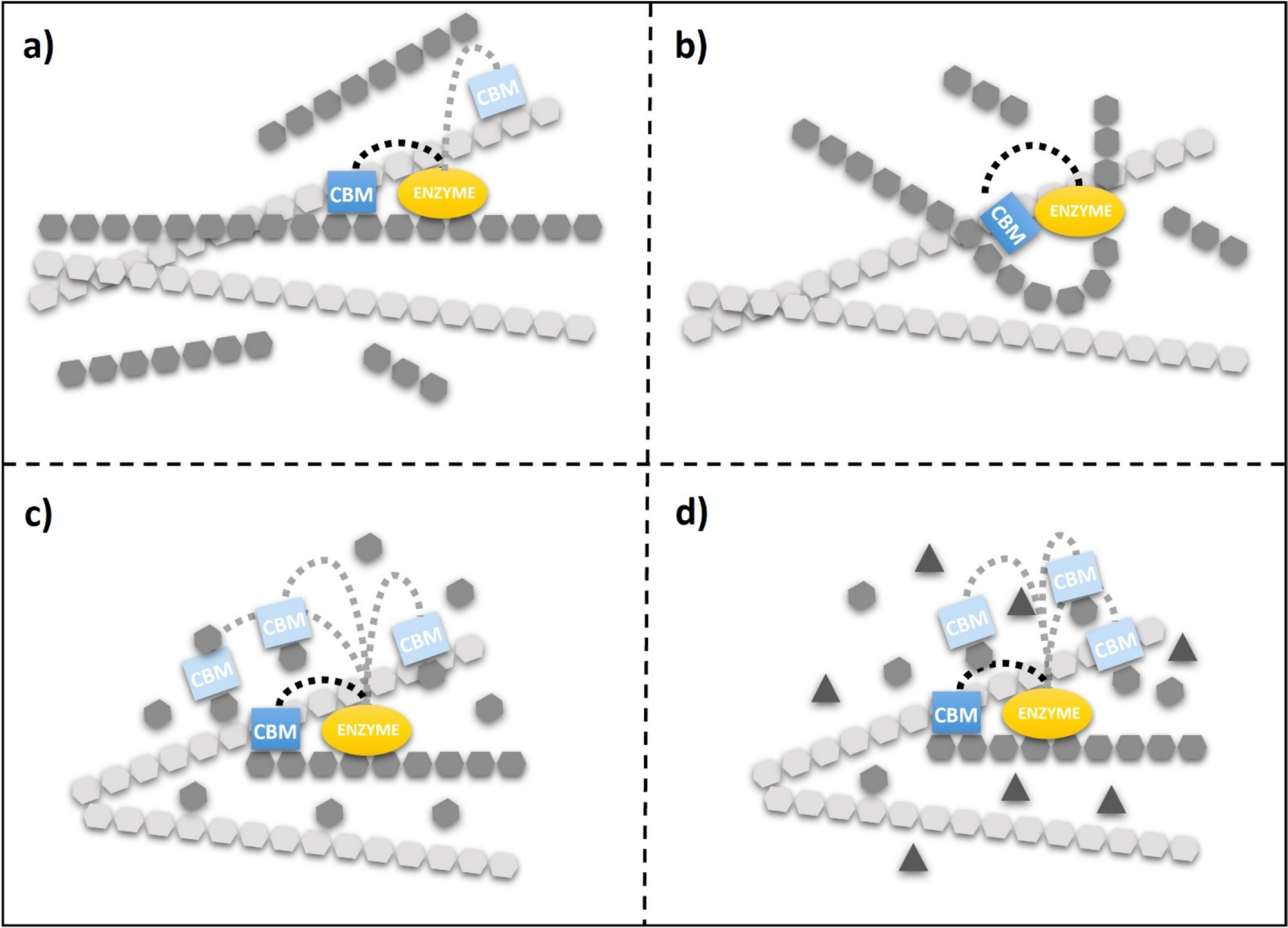
Functional significance of carbohydrate binding modules (CBMs). A schematic description of the putative functions of CBMs is presented:  **a** targeting of the enzyme towards its substrate, **b** guidance of the substrate towards the active site groove, **c** improving processivity, **d** specificity specificity

Another approach to enhance enzyme activity by chimeric enzymes is to add specific modules to single-domain enzymes. Such is the case for carbohydrate-binding modules (CBMs), which have a high binding affinity for polysaccharides. Although these modules are not domains, they have a specific fold and enhance enzymatic activity. CBMs can be found in the N- or C-terminal domain of glycosyl hydrolases (GH) and are connected to the catalytic domain by a repeat linker of threonine-proline residues [35]. CBMs increase the activity of enzymes by facilitating the association of the enzyme with the substrate; therefore, the local substrate concentration at the active site increases (Fig. 3) [36]. CBMs have different folds, for example, OB-fold, β-trefoil, lectin-like, and β-sandwich, the latter being the most common. However, these folds are not predictive of their function [37].

Furtado et al. (2015) fused the xyloglucan-specific CBM44 from *C. thermocellum* with the GH12 XEGA from *Aspergillus niveus* (EC 3.2.1.151). This chimeric enzyme (CBM44-XEGA) increased xyloglucan hydrolysis by 30%, and the catalytic efficiency showed a kcat 1.25 times higher than the parent enzyme XEGA. The presence of the CBM did not modify the hydrolysis product profile; therefore, this module does not affect the enzyme catalytic mechanism [38]. The chimeric XynB-CBM2b xylanase was formed by fusing a *Streptomyces thermoviolaceus* STX-II family 2b carbohydrate-binding module to the carboxyl terminus of XynB, a thermostable single-domain xylanase from *Thermotoga maritima* family 10. XynB-CBM2b showed a 1.7-fold higher kcat against soluble birchwood xylan than the wild-type enzyme [39]. In another example, the chimeric enzyme XYN-TmCBM9-1_2 is a fusion of TmCBM9-1_2 (CBM from *T. maritima*) and the xylanase XYN from *A. niger*. This chimeric enzyme resulted in a 4.2-fold increase in xylanase activity on insoluble oat-spelled xylan compared to soluble birchwood xylan [40].

A similar effect was reported when fusing CBM (type A and B) to the end of the N- or C-terminal domain of the gluco-oligosaccharide oxidase (EC 1.1.99) from *Sarocladium strictum* CBS 346.70 (GOOX). All enzymes with CBM at the N-terminus and one at the C-terminus showed higher catalytic activity on the oligosaccharides tested than on wild-type GOOX, suggesting a positive conformational change. Unchanged Km values confirmed that the fused CBMs did not compete with GOOX subsites for oligosaccharide binding. The addition of type-B CBM increased the affinity of GOOX towards soluble polysaccharides, including β-glucan, glucomannan, and xyloglucan. These chimeric enzymes increased the catalytic activity (kcat) on cellotetraose by 50%, glucomannan by 30%, and amorphous (insoluble crystal) cellulose by more than 50%. The addition of CBM at the end of the N- or C-terminal domain did not affect the catalytic activity [41].

Chimeric enzymes using CBMs have also allowed modification of the enzyme’s substrate specificity. Han et al. (2013) constructed a chimeric enzyme formed by the cyclodextrin glycosyltransferase (EC 2.4.1.19) from *Paenibacillus macerans* (CGTase) and the CBM of α-amylase from *Alkalimonas amylolytica* (CBMAmy). The chimeric enzyme synthesized 2-OD-glucopyranosyl-L-ascorbic acid (AA-2G), an ascorbic acid derivative produced from soluble starch as a cheap and easily soluble glucosyl donor. Under optimal conditions, the yield of AA-2G produced with CGT-CBMAmy was 5.94-fold higher than the yield obtained with wild-type CGTase. The authors suggest that the enhancement of soluble starch specificity may be related to changes in substrate binding capacity and substrate binding sites between the CBM and the starch granule. A kinetic reaction model carried out by the authors confirmed that this effect is due to the increased specificity of the soluble starch provided by CBMAmy [42]. These examples demonstrate that generating CBM-linked chimeric enzymes improves the catalytic activity. These and other recent and promising examples are summarized in Table 2.

**Table 2:** Chimeric enzymes with improved catalytic properties by addition of a CBM.

Enzyme	EC number	Organism	CBM added	CBM Origin	Enhanced activity properties	Refs.
Gluco-oligosaccharide oxidase GOOX	EC 1.1.3	*Sarocladium strictum* CBS 346.70	Type-A CtCBM3 Type-B CtCBM11 Type-B CtCBM44	*Clostridium thermocellum* CipA *C. thermocellum* Lic26A-Cel5E *C. thermocellum* Cel9D-Cel44A	1.2–1.4-fold enhanced activity on linear β-glucans. Increase in kcat over cellotetraose (50%), glucomannan (30%), and amorphous cellulose (50%).	[41]
Gluco-oligosaccharide oxidase GOOX-VN	EC 1.1.3	*Sarocladium strictum* CBS 346.70	CBM22-2	*Clostridium thermocellum*	CBM22-2 at the N-terminal of GOOX-VN increased catalytic activity on mono- and oligo-saccharides by 2–3 fold.	[43]
Cyclodextrin glycosyltransferase CGTase	EC 2.4.1.19	*Paenibacillus macerans*	CBMAmy	*Alkalimonas amylolytica* α-amylase	CGT-CBMAmy yield was 5.94-fold compared to wild-type CGTase when using soluble starch as substrate.	[42]
Cutinase	EC 3.1.1.74	*Thermobifida fusca*	CBMCel6A CBMCenA	*T. fusca* cellulase Cel6A *Cellulomonas fimi* cellulase CenA	Binding activity on cotton fiber was increased by 2% for cutinase-CBMCel6A and 28% for cutinase-CBMCenA compared to wild-type cutinase.	[44]
Cellobiohydrolases Cel7A	EC 3.2.1.	*Talaromyces emersonii*	CBM1 CBM2 CBM3 cellulosomal-scaffolding protein (1NBC)	*T. reesei* Cel7A *C. fimi* *Clostridium thermocellum*	Purified chimeric enzymes bound to cellulose better than the catalytic module alone. They proved to have high thermal stability, with temperatures between 72 and 77 °C.	[45]
Chitinase D SpChiD	EC 3.2.1.14	*Serratia proteamaculans*	Polycystic kidney disease (PKD) domain Chitin binding protein 21 (CBP21)	ND	ChiD + PKD and ChiD + CBP have a larger catalytic efficiency, 1.9 and 1.3-fold respectively than wild type enzyme.	[46]
Endoglucanase Cel5A	EC:3.2.1.4	Derived from the metagenomic library of vermicompost.	CBM6	*Saccharophagus degradans* endoglucanase Cel5H	Cel5A_2R2-CBM6 showed 7-fold higher activity and a significantly higher binding affinity towards avicel than the parent Cel5A_2R2.	[47]
Thermophilic endoglucanases Cel9A Cel5A	EC 3.2.1.4	*Alicyclobacillus acidocaldarius* *Thermotoga maritima*	CBM2a	*Thermomonospora fusca*. thermophilic exoglucanase E3	Increases cellulase activity by up to 3-fold on insoluble cellulosic substrates as compared to wild type.	[48]
Hybrid microbial cellulase CEL-HYB1	EC 3.2.1.4	*Hordeum vulgare* L. cv. Golden Promise	CBM of the tomato	*Solanum lycopersicum* SlCel9C1 cellulase	CEL-HYB1-CBM demonstrated a 4-fold higher exo-glucanase activity and 6-fold higher catalytic efficiency for the hydrolysis of crystalline cellulose than the wild-type CEL-HYB1.	[49]
β-mannanase AuMan5A	EC 3.2.1.78	*Aspergillus usamii* YL-01-78	CBM1	*Trichoderma reesei* cellobiohydrolase I (TrCBH I)	The cellulose-binding capacity of the reAuMan5A-CBM was up to 92.3%, whereas reAuMan5A was unable to bind CBM, and Km values were 0.66 mg/mL and 1.36 mg/mL, respectively.	[50]
D-xylose isomerase	EC 5.3.1.5	*Thermotoga neapolitana* 5068 (TNXI)	Chitin-binding domain (CBD)	*Pyrococcus furiosus* chitinase (PF1233)	kcat for CBD-TNXI increased 4-fold compared to wild-type TNXI.	[51]

*ND* Not described

## Construction of chimeric enzymes: combining and modifying the specificity

Generating new biocatalysts is an imperative issue that innovative areas need to obtain new compounds and improve bioprocesses. An alternative to achieve this goal is to design biocatalysts by modifying or combining the specificity of the enzymes by chimerization.

Domain combinations may modify the specificity of enzymes and even confer novel biosynthetic activities, such as in the case of the chimeric 6-methyl salicylic acid synthase (6MSAS) from *A. terreus*. 6MSAS catalyzes the synthesis of 6-methyl salicylic acid (6MSA); it is a multidomain enzyme consisting of a keto synthase (KS), acyltransferase (AT), dehydratase (DH), ketoreductase (KR) and acyl carrier protein (ACP) domains. The construction of a chimeric 6MSAS enzyme replacing the ACP and methyltransferase (MT) domains by those of the polyketide synthase (PKS) from *Pseudallescheria boydii*, resulted in a specificity change, synthesizing an entirely different compound, the 2-hydroxy-2-(propane-2-yl) cyclobutane-1,3-dione. Although the exact role of the fused domains remains to be demonstrated, this example highlights the potential of the chimerization strategy to modify enzyme specificity [52].

Sometimes, combining activities changes the enzyme activity or specificity and improves other properties. The union of a β-xylanase (26 kDa) from *B. subtilis* to the catalytic domain of a β-xylosidase (60 kDa) from *B. subtilis* by a linker of 26 residues from *Thermotoga petrophila* resulted in a bifunctional chimera with improved thermostability. The optimum temperature for β-xylosidase activity was increased by 5.3 °C and retained 80% activity after 45 h-incubation at 45 °C, while the wild-type enzyme retained only 30%. Further, the chimera catalyzed the production of 3-fold more xylose than parental enzymes. These changes are due to the oligomerization of the chimeric enzyme [29]. For another example, Adlakha et al. (2011) constructed four chimeric enzymes with β-1,4-endoglucanase (Endo5A) and β-1,4-endoxylanase (Xyl11D) from the *Paenibacillus* sp. ICGEB2008. These chimeric enzymes were built by changing the order of the enzymes and using a glycine-serine linker. The chimeric enzymes showed 0.5- to 1.6-fold and 1.3- to 2.3-fold higher enzyme activity than Xyl11D and Endo5A, respectively. One of these chimeras showed the highest endoglucanase (1070 U µmol^−1^) and xylanase (899 U µmol^−1^) activities. The authors mention that the orientation and the linker are important for the optimal activity of both enzymes, suggesting that the reaction yield was increased by the proximity of the catalytic centers in the chimeric enzyme [53]. Although domain combining is a promising strategy to increase enzyme activity, enzyme activity is sometimes negatively affected [54] or does not significantly increase compared to parental enzymes [55].

Until now, multifunctional chimeric biocatalysts have been used in bioprocesses for the bioconversion of agricultural by-products, the biotransformation of xenobiotics, and the production of food ingredients [56]. For example, Furtado et al. (2013) developed a chimeric enzyme with laccase and glucanase activities. The chimeric enzyme consisted of *B. subtilis* CotA laccase (EC 1.10.3.2) and *B. subtilis* β-1,3–1,4-glucanase BglS (EC 3.2.1.73). Laccase breaks down lignin by catalyzing the oxidation of aromatic compounds with a simultaneous reduction of oxygen to water. On the other hand, glucanase hydrolyzes the β-glucans of hemicellulose. The hydrolytic activity of the chimeric enzyme was 20% higher in sugarcane bagasse compared to equimolar mixtures of wild-type enzymes. The authors associated this increased hydrolytic activity with the proximity between domains in the chimera, resulting in higher reaction rates in sequential reactions and improved substrate binding. Molecular dynamics simulations determined that forming an interface between domains enhanced the accessibility of substrates to the catalytic domain [57].

Chimeric enzymes can be constructed by two enzymes catalyzing consecutive reactions. In this case, one enzyme converts a substrate to a product, which the second enzyme uses as a substrate to synthesize a final product. One example is the chimeric enzyme DXSR, which was constructed to develop a one-step process for producing isomalto-oligosaccharides (IMO). DXSR is a chimeric enzyme that contains an endo-dextranase from *Arthrobacter oxydans* and an α- [1–6] dextransucrase from *L. mesenteroides* B-1299CB4. DXSR had a 150% increase in endo-dextranase activity and a 98% decrease in dextransucrase activity compared to the individual enzymes. However, DXSR catalyzed a 30-fold higher production of IMO than an equal activity mixture of the wild-type enzymes. DXSR can be applied to produce IMO from sucrose by a one-step reaction, and the size of IMOs can be controlled by modulating sucrose concentration and overall reaction time [58].

Constructing chimeric enzymes is a strategy that can be used to evaluate other activities in preparing metabolites and other compounds of interest for medicinal chemistry. To identify activities for the transformation of drug molecules, Kulig et al. (2015) constructed fifteen chimeric enzymes which comprised several *Rhodococcus jostii* cytochrome P450 heme domain with the *Rhodococcus sp* P450 reductase domain (RhfRED) of cytochrome P450Rhf. Strains expressing chimeric RhfRED enzymes were exposed to commercially available drugs and revealed different activities commensurate with P450-catalyzed hydroxylation and demethylation reactions. One of these chimeric enzymes catalyzed the N-demethylation of diltiazem and imipramine. Characterization of this enzyme revealed a 63% conversion of imipramine to the N-demethylated product [59].

Another strategy used to improve biocatalysts is the development of libraries to bio-convert hydrophobic and xenobiotic compounds. An example of the combination of activities by domain binding is the work reported by Corrado et al. (2018). The authors generated a chimeric styrene monooxygenase (Fus-SMO) by fusing, through a flexible linker, the enzymes StyA and StyB that belong to the styrene monooxygenase (SMO) system from *Pseudomonas sp*. StyB catalyzes the reduction of FAD to FADH_2_ at the expense of NADH, while StyA utilizes FADH_2_ and O_2_ to generate FAD-OOH, the epoxidizing agent. In this case, the epoxidation activity of Fus-SMO was up to three times higher than the two-component StyA/StyB (1:1, molar ratio) system.

Furthermore, the solubility of Fus-SMO was higher than that of heterologous StyB expressed in *E. coli*. This work shows that combining activities could improve the expression levels of the reductase and epoxidase units [60]. The examples mentioned above illustrate the possibility of improving the catalytic activity of enzymes and generating new catalytic activities using chimeric enzymes. Table 3 shows more examples of the benefits the chimerization strategy of combining activities can confer to obtain better biocatalysts.

**Table 3:** Synthetic chimeric enzymes that merged two activities

Fused Enzymes	EC numbers	Organism	Improved Property	Refs.
Geranylgeranyl diphosphate synthase (GPPS) and phytoene synthase (PSY)	2.5.1.29 and 2.5.1.32	*Arabidopsis thaliana*	Removed enzymatic competition for geranylgeranyl diphosphate and increased the carotenoid content by 50%.	[61]
Polyethylene terephthalate hydrolase (PETase) and mono(2-hydroxyethyl) terephthalate hydrolase (MHETase)	3.1.1.101 and 3.1.1.102	*Ideonella sakaiensis*	Improved MHETase and PETase activities increased PET degradation and MHET hydrolysis rates.	[62]
Lipase (Lip) and cutinase (Cut)	3.1.1.3 and 3.1.1.74	*Thermomyces lanuginosus* and *Thielavia terrestris*	Enhanced lipase and cutinase activities by 127% and 210% than their parental enzymes.	[63]
Endoglucanase (CtGH5) mutant F194A and β-1,4-glucosidase (CtGH1)	3.2.1.4 and 3.2.1.21	*Clostridium thermocellum*	Enhanced enzyme activity, thermostability and structural integrity of both enzymes.	[64]
Mutanase and dextranase	3.2.1.59 and 3.2.1.11	*Paenibacillus humicus* NA1123 and *Streptococcus mutans* ATCC 25,175	4.1 times more effective at glucan inhibition of biofilm formation than a mixture of dextranase and mutanase.	[65]
Glucuronan lyase (TrGL) and a chitinase (ThCHIT42)	4.2.2.14 and 3.2.1.14	*Trichoderma sp.*	Increased the velocity of glucuronan lyase	[66]

## Design and methods for constructing multidomain enzymes

The molecular biology methods established to manufacture end-to-end fusion enzymes allow the linkage of two or more domains at the N- or C-terminus. These methods include overlapping extension polymerase chain reaction (OE-PCR), restriction cloning, and recombination approaches. OE-PCR is now the mainstay of gene fusions, allowing exact manipulation of DNA and modification of the ends of DNA fragments for downstream processing. This method generally requires a pair of primers for each gene. The forward primer encloses a restriction enzyme cleavage site necessary for further ligation into an expression vector. The reverse primer presents a region complementary to the second gene of the construct. The primers of the second gene are generated oppositely. OE-PCR allows the two PCR products obtained above to bind freely, regardless of restriction sites. Like OE-PCR, restriction cloning, homologous recombination approaches, and Golden Gate cloning help combine multiple genes into a single vector system [67–72].

In contrast to the above techniques, the LE (*LguI/Eco81I*)-cloning system has been specially designed to generate end-to-end multi-fusion enzymes in a continuously growing vector system, facilitating their arrangement of different domain combinations to establish which specific grouping of domains exhibits an improved activity [73].

Occasionally, the proximity of two domains can result in unfavorable folding, resulting in the loss of activity of one or both catalytic domains. In these cases, adding linker sequences (subdivided into three classes: flexible, rigid, and in vivo cleavable linkers) may allow for better conformation, stability, and autonomous actions of each functional domain in a fusion protein [74].

Linkers could provide mobility and flexibility between domains, factors essential to the efficient biocatalytic function of a chimeric enzyme. Xue et al. (2009) constructed a trifunctional enzyme to degrade agricultural by-products effectively. This chimeric enzyme was composed of xylosidase-arabinosidase (Xar, EC 3.2.1.55) from *Thermoanaerobacter ethanolicus* and xylanase (XynA, EC 3.2.1.8) from *Thermomyces lanuginosu*. The chimeric enzyme decreased arabinosidase-xylosidase activity despite increased xylanase activity [75]. However, the chimeric enzyme was improved by adding a glycine-rich linker [76]. In the same way, Lu et al. (2006) reported the construction of a chimeric enzyme with xylanase and β-1,3 − 1,4-glucanase from *B. amyloliquefaciens* by end-to-end gene fusion. Although this chimeric enzyme decreased xylanase activity, adding a glycine-rich linker between the subunits enhanced xylanase and glucanase activity [77, 78].

## Concluding remarks

In nature, domain acquisition is an adaptive evolutionary process that expands and modulates enzyme properties. This process has been replicated in all types of enzymes so that chimeric enzymes are present in the seven enzyme classes-: oxidoreductases, transferases, hydrolases, lyases, isomerases, ligases, and translocases. In some instances, domain acquisition is responsible for the modulation of essential properties from the biocatalysis point of view, such as catalytic activity and stability. It may also affect substrate recognition and specificity. Thus, creating multidomain chimeric enzymes may be another strategy for biocatalysis improvement, which could potentiate the local effects of single-point mutation obtained through rational or directed evolution, expanding the toolbox of protein engineering.

However, at the moment, the role of domain-domain interactions in enzyme catalysis still needs to be understood entirely. Hence, creating improved enzymes through this strategy is an attractive yet undeveloped alternative. Successful examples are based on reasonable expectations, such as replacing one mesophile domain for an extremophile domain, as in the case of thermostable multidomain chimeric enzymes. More subtle properties, such as substrate specificity, are more difficult to rationalize, manipulate, and predict for multidomain chimeric enzymes. Recent advances *in silico* approaches, such as computational simulation of proteins and the inclusion of powerful tools such as artificial intelligence for the structural study of proteins, may provide a massive leap in our comprehension of complex protein interactions in a short time.

## Data Availability

The datasets analyzed during the current study are available from the corresponding author upon reasonable request.

## References

[1] Wu S, Snajdrova R, Moore JC, Baldenius K, Bornscheuer UT (2021) Biocatalysis: enzymatic synthesis for Industrial Applications. Angew Chemie - Int Ed 60(1):88–119 10.1002/anie.202006648 PMC7818486 32558088

[2] Moore AD, Björklund ÅK, Ekman D, Bornberg-Bauer E, Elofsson A (2008) Arrangements in the modular evolution of proteins. Trends Biochem Sci 33(9):444–451 18656364 10.1016/j.tibs.2008.05.008

[3] Ekman D, Björklund ÅK, Frey-Skött J, Elofsson A (2005) Multi-domain proteins in the three kingdoms of life: orphan domains and other unassigned regions. J Mol Biol 348(1):231–243 15808866 10.1016/j.jmb.2005.02.007

[4] Narhi LO, Fulco AJ (1986) Characterization of a catalytically self-sufficient 119,000-dalton cytochrome P-450 monooxygenase induced by barbiturates in *Bacillus megaterium*. J Biol Chem 261(16):7160–7169 3086309

[5] Munro AW, Girvan HM, McLean KJ (2007) Cytochrome P450-redox partner fusion enzymes. Biochim Biophys Acta - Gen Subj 1770(3):345–359 10.1016/j.bbagen.2006.08.018 17023115

[6] Sadeghi SJ, Gilardi G (2013) Chimeric P450 enzymes: activity of artificial redox fusions driven by different reductases for biotechnological applications. Biotechnol Appl Biochem 60:102–110 23586997 10.1002/bab.1086

[7] Olivares-Illana V, López-Munguía A, Olvera C (2003) Molecular characterization of inulosucrase from *Leuconostoc citreum*: a fructosyltransferase within a glucosyltransferase. J Bacteriol 185(12):3606–3612 12775698 10.1128/JB.185.12.3606-3612.2003 PMC156214

[8] Del Moral S, Olvera C, Rodriguez ME, Munguia AL (2008) Functional role of the additional domains in inulosucrase (IslA) from *Leuconostoc citreum* CW28. BMC Biochem 9(1):6 18237396 10.1186/1471-2091-9-6 PMC2270844

[9] Morales-Arrieta S, Rodríguez ME, Segovia L, López-Munguía A, Olvera-Carranza C (2006) Identification and functional characterization of levS, a gene encoding for a levansucrase from *Leuconostoc mesenteroides* NRRL B-512 F. Gene 376(1–2):59–67 16632262 10.1016/j.gene.2006.02.007

[10] Olvera C, Centeno-Leija S, López-Munguía A (2007) Structural and functional features of fructansucrases present in *Leuconostoc mesenteroides* ATCC 8293. Antonie Van Leeuwenhoek. Int J Gen Mol Microbiol 92(1):11–20 10.1007/s10482-006-9128-0 17109058

[11] García-Paz F, de Martínez-Bahena M, Olvera S (2022) Structure–function relationship studies of multidomain levansucrases from *Leuconostocaceae* family. Microorganisms 10(5):889 35630334 10.3390/microorganisms10050889 PMC9142893

[12] Pan S, Ding N, Ren J, Gu Z, Li C, Hong Y et al (2017) Maltooligosaccharide-forming amylase: characteristics, preparation, and application. Biotechnol Adv 35(5):619–632 28457999 10.1016/j.biotechadv.2017.04.004

[13] Ding N, Zhao B, Han X, Li C, Gu Z, Li Z (2022) Starch-binding domain modulates the specificity of maltopentaose production at moderate temperatures. J Agric Food Chem 70(29):9057–9065 35829707 10.1021/acs.jafc.2c03031

[14] Tamaru Y, Doi RH (2001) Pectate lyase A, an enzymatic subunit of the *Clostridium cellulovorans* cellulosome. Proc Natl Acad Sci U S A 98(7):4125–4129 11259664 10.1073/pnas.071045598 PMC31190

[15] Martinez Cuesta S, Furnham N, Rahman SA, Sillitoe I, Thornton JM (2014) The evolution of enzyme function in the isomerases. Curr Opin Struct Biol 26(1):121–130 25000289 10.1016/j.sbi.2014.06.002 PMC4139412

[16] Wilkinson B, Gilbert HF (2004) Protein disulfide isomerase. Biochim Biophys Acta - Proteins Proteom 1699(1–2):35–44 10.1016/j.bbapap.2004.02.017 15158710

[17] Darby NJ, Penka E, Vincentelli R (1998) The multi-domain structure of protein disulfide isomerase is essential for high catalytic efficiency. J Mol Biol 276(1):239–247 9514721 10.1006/jmbi.1997.1504

[18] Anand R, Hoskins AA, Stubbe JA, Ealick SE (2004) Domain organization of *Salmonella typhimurium* formylglycinamide ribonucleotide amidotransferase revealed by X-ray crystallography. Biochemistry 43(32):10328–10342 15301531 10.1021/bi0491301

[19] Xu X, Shi H, Gong X, Gao Y, Zhang X, Xiang S (2020) Structural insights into sodium transport by the oxaloacetate decarboxylase sodium pump. Elife 9:1–53 10.7554/eLife.53853 PMC7274780 32459174

[20] Studer R, Dahinden P, Wang WW, Auchli Y, Li XD, Dimroth P (2007) Crystal structure of the Carboxyltransferase Domain of the Oxaloacetate Decarboxylase na + pump from *Vibrio cholerae*. J Mol Biol 367(2):547–557 17270211 10.1016/j.jmb.2006.12.035

[21] Dahinden P, Pos KM, Dimroth P (2005) Identification of a domain in the α-subunit of the oxaloacetate decarboxylase na + pump that accomplishes complex formation with the γ-subunit. FEBS J 272(3):846–855 15670164 10.1111/j.1742-4658.2004.04524.x

[22] Illanes A, Cauerhff A, Wilson L, Castro GR (2012) Recent trends in biocatalysis engineering. Bioresour Technol 115:48–57 22424920 10.1016/j.biortech.2011.12.050

[23] Sun JY, Liu MQ, Xu YL, Xu ZR, Pan L, Gao H (2005) Improvement of the thermostability and catalytic activity of a mesophilic family 11 xylanase by N-terminus replacement. Protein Expr Purif 42(1):122–130 15939297 10.1016/j.pep.2005.03.009

[24] Yin X, Li JF, Wang JQ, Tang CD, Wu MC (2013) Enhanced thermostability of a mesophilic xylanase by N-terminal replacement designed by molecular dynamics simulation. J Sci Food Agric 93(12):3016–3023 23512640 10.1002/jsfa.6134

[25] Sorrentino I, Giardina P, Piscitelli A (2019) Development of a biosensing platform based on a laccase-hydrophobin chimera. Appl Microbiol Biotechnol 103(7):3061–3071 30783720 10.1007/s00253-019-09678-2

[26] Gilmore SP, Lillington SP, Haitjema CH, de Groot R, O’Malley MA (2020) Designing chimeric enzymes inspired by fungal cellulosomes. Synth Syst Biotechnol 5(1):23–32 32083193 10.1016/j.synbio.2020.01.003 PMC7015840

[27] Chen Z, Wang L, Shen Y, Hu D, Zhou L, Lu F et al (2022) Improving thermostability of Chimeric Enzymes Generated by Domain Shuffling between two different original glucoamylases. Front Bioeng Biotechnol 10(April):1–11 10.3389/fbioe.2022.881421 PMC9017332 35449593

[28] Song L, Dumon C, Siguier B, André I, Eneyskaya E, Kulminskaya A et al (2014) Impact of an N-terminal extension on the stability and activity of the GH11 xylanase from *Thermobacillus xylanilyticus*. J Biotechnol 174(1):64–72 24440633 10.1016/j.jbiotec.2014.01.004

[29] Diogo JA, Hoffmam ZB, Zanphorlin LM, Cota J, Machado CB, Wolf LD et al (2015) Development of a chimeric hemicellulase to enhance the xylose production and thermotolerance. Enzyme Microb Technol 69:31–37 25640722 10.1016/j.enzmictec.2014.11.006

[30] Crum MA, Park JM, Sewell BT, Benedik MJ (2015) C-terminal hybrid mutant of *Bacillus pumilus* cyanide dihydratase dramatically enhances thermal stability and pH tolerance by reinforcing oligomerization. J Appl Microbiol 118(4):881–889 25597384 10.1111/jam.12754

[31] Cui Y, Cui W, Liu Z, Zhou L, Kobayashi M, Zhou Z (2014) Improvement of stability of nitrile hydratase via protein fragment swapping. Biochem Biophys Res Commun 450(1):401–408 24944015 10.1016/j.bbrc.2014.05.127

[32] Nick Pace C, Martin Scholtz J, Grimsley GR (2014) Forces stabilizing proteins. FEBS Lett 588(14):2177–2184 24846139 10.1016/j.febslet.2014.05.006 PMC4116631

[33] Branchini BR, Southworth TL, Fontaine DM, Davis AL, Behney CE, Murtiashaw MH (2014) A *Photinus pyralis* and *Luciola italica* chimeric firefly luciferase produces enhanced bioluminescence. Biochemistry 53(40):6287–6289 25264115 10.1021/bi501202u

[34] Olvera C, Centeno-Leija S, Ruiz-Leyva P, López-Munguía A (2012) Design of chimeric levansucrases with improved transglycosylation activity. Appl Environ Microbiol 78(6):1820–1825 22247149 10.1128/AEM.07222-11 PMC3298123

[35] Shoseyov O, Shani Z, Levy I (2006) Carbohydrate binding modules: biochemical properties and Novel Applications. Microbiol Mol Biol Rev 70(2):283–295 16760304 10.1128/MMBR.00028-05 PMC1489539

[36] Bashton M, Chothia C (2007) The generation of new protein functions by the combination of domains. Structure 15(1):85–99 17223535 10.1016/j.str.2006.11.009

[37] Boraston AB, Bolam DN, Gilbert HJ, Davies GJ (2004) Carbohydrate-binding modules: fine-tuning polysaccharide recognition. Biochem J 382(3):769–781 15214846 10.1042/BJ20040892 PMC1133952

[38] Furtado GP, Santos CR, Cordeiro RL, Ribeiro LF, de Moraes LAB, Damásio ARL et al (2015) Enhanced xyloglucan-specific endo-β-1,4-glucanase efficiency in an engineered CBM44-XegA chimera. Appl Microbiol Biotechnol 99(12):5095–5107 25605422 10.1007/s00253-014-6324-0

[39] Kittur FS, Mangala SL, Rus’d AA, Kitaoka M, Tsujibo H, Hayashi K (2003) Fusion of family 2b carbohydrate-binding module increases the catalytic activity of a xylanase from *Thermotoga maritima* to soluble xylan. FEBS Lett 549(1–3):147–151 12914941 10.1016/s0014-5793(03)00803-2

[40] Liu L, Cheng J, Chen H, Li X, Wang S, Song A et al (2011) Directed evolution of a mesophilic fungal xylanase by fusion of a thermophilic bacterial carbohydrate-binding module. Process Biochem 46(1):395–398

[41] Foumani M, Vuong TV, MacCormick B, Master ER (2015) Enhanced polysaccharide binding and activity on Linear β-Glucans through addition of carbohydrate-binding modules to either terminus of a glucooligosaccharide oxidase. PLoS ONE 10(5):e0125398 25932926 10.1371/journal.pone.0125398 PMC4416756

[42] Han R, Li J, Shin HD, Chen RR, Du G, Liu L et al (2013) Carbohydrate-binding module-cyclodextrin glycosyltransferase fusion enables efficient synthesis of 2-O-d-glucopyranosyl-l-ascorbic acid with soluble starch as the glycosyl donor. Appl Environ Microbiol 79(10):3234–3240 23503312 10.1128/AEM.00363-13 PMC3685265

[43] Vuong TV, Master ER (2014) Fusion of a xylan-binding module to gluco- oligosaccharide oxidase increases activity and promotes stable immobilization. PLoS ONE 9(4):e95170 24736604 10.1371/journal.pone.0095170 PMC3988151

[44] Zhang Y, Chen S, Xu M, Cavoco-Paulo AP, Wu J, Chen J (2010) Characterization of *Thermobifida fusca* cutinase-carbohydrate-binding module fusion proteins and their potential application in bioscouring∇. Appl Environ Microbiol 76(20):6870–6876 20729325 10.1128/AEM.00896-10 PMC2953015

[45] Voutilainen SP, Nurmi-Rantala S, Penttilä M, Koivula A (2014) Engineering chimeric thermostable GH7 cellobiohydrolases in *Saccharomyces cerevisiae*. Appl Microbiol Biotechnol 98:2991–3001 23974371 10.1007/s00253-013-5177-2

[46] Madhuprakash J, El Gueddari NE, Moerschbacher BM, Podile AR (2015) Catalytic efficiency of chitinase-D on insoluble chitinous substrates was improved by fusing auxiliary domains. PLoS ONE 10(1):e0116823 25615694 10.1371/journal.pone.0116823 PMC4304778

[47] Telke AA, Zhuang N, Ghatge SS, Lee SH, Ali Shah A, Khan H et al (2013) Engineering of Family-5 glycoside hydrolase (Cel5A) from an uncultured bacterium for efficient hydrolysis of cellulosic substrates. PLoS ONE 8(6):e65727 23785445 10.1371/journal.pone.0065727 PMC3681849

[48] Reyes-Ortiz V, Heins RA, Cheng G, Kim EY, Vernon BC, Elandt RB et al (2013) Addition of a carbohydrate-binding module enhances cellulase penetration into cellulose substrates. Biotechnol Biofuels 6(1):93 23819686 10.1186/1754-6834-6-93 PMC3716932

[49] Byrt CS, Cahyanegara R, Grof CPL (2012) Plant carbohydrate binding module enhances activity of hybrid microbial cellulase enzyme. Front Plant Sci 3:254 23181066 10.3389/fpls.2012.00254 PMC3501001

[50] Tang CD, Li JF, Wei XH, Min R, Gao SJ, Wang JQ et al (2013) Fusing a carbohydrate-binding Module into the *aspergillus usamii* β-Mannanase to improve its thermostability and cellulose-binding capacity by in Silico Design. PLoS ONE 8(5):e64766 23741390 10.1371/journal.pone.0064766 PMC3669383

[51] Harris JM, Epting KL, Kelly RM (2010) N-Terminal fusion of a hyperthermophilic chitin-binding domain to xylose isomerase from *Thermotoga neapolitana* enhances kinetics and thermostability of both free and immobilized enzymes. Biotechnol Prog 26(4):993–1000 20730758 10.1002/btpr.416 PMC3711014

[52] Liao JL, Pang KL, Sun GH, Pai TW, Hsu PH, Lin JS et al (2019) Chimeric 6-methylsalicylic acid synthase with domains of acyl carrier protein and methyltransferase from *Pseudallescheria boydii* shows novel biosynthetic activity. Microb Biotechnol 12(5):920–931 31199579 10.1111/1751-7915.13445 PMC6681407

[53] Adlakha N, Rajagopa R, Kumar S, Reddy VS, Yazdani SS (2011) Synthesis and characterization of chimeric proteins based on cellulase and xylanase from an insect gut bacterium. Appl Environ Microbiol 77(14):4859–4866 21642416 10.1128/AEM.02808-10 PMC3147366

[54] Wang TT, Deng JQ, Chen LZ, Sun L, Wang FS, Ling PX et al (2020) The second member of the bacterial UDP-N-acetyl-D-glucosamine:heparosan alpha-1, 4-N-acetyl-D-glucosaminyltransferase superfamily: GaKfiA from *Gallibacterium anatis*. Int J Biol Macromol 147:170–176 31923511 10.1016/j.ijbiomac.2020.01.016

[55] Cota J, Oliveira LC, Damásio ARL, Citadini AP, Hoffmam ZB, Alvarez TM et al (2013) Assembling a xylanase-lichenase chimera through all-atom molecular dynamics simulations. Biochim Biophys Acta - Proteins Proteom 1834(8):1492–1500 10.1016/j.bbapap.2013.02.030 23459129

[56] Yu K, Liu C, Kim BG, Lee DY (2015) Synthetic fusion protein design and applications. Biotechnol Adv 33(1):155–164 25450191 10.1016/j.biotechadv.2014.11.005

[57] Furtado GP, Ribeiro LF, Lourenzoni MR, Ward RJ (2013) A designed bifunctional laccase/β-1,3 – 1,4-glucanase enzyme shows synergistic sugar release from milled sugarcane bagasse. Protein Eng Des Sel 26(1):15–23 23012443 10.1093/protein/gzs057

[58] Kim YM, Seo MY, Kang HK, Atsuo K, Kim D (2009) Construction of a fusion enzyme of dextransucrase and dextranase: application for one-step synthesis of isomalto-oligosaccharides. Enzyme Microb Technol 44(3):159–164

[59] Kulig JK, Spandolf C, Hyde R, Ruzzini AC, Eltis LD, Grönberg G et al (2015) A P450 fusion library of heme domains from *Rhodococcus jostii* RHA1 and its evaluation for the biotransformation of drug molecules. Bioorg Med Chem 23(17):5603–5609 26234905 10.1016/j.bmc.2015.07.025

[60] Corrado ML, Knaus T, Mutti FG (2018) A chimeric styrene monooxygenase with increased efficiency in Asymmetric Biocatalytic Epoxidation. ChemBioChem 19(7):679–686 29378090 10.1002/cbic.201700653 PMC5900736

[61] Camagna M, Grundmann A, Bar C, Koschmieder J, Beyer P, Welsch R (2019) Enzyme fusion removes competition for geranylgeranyl diphosphate in carotenogenesis. Plant Physiol 179(3):1013–1027 30309967 10.1104/pp.18.01026 PMC6393812

[62] Knott BC, Erickson E, Allen MD, Gado JE, Graham R, Kearns FL et al (2020) Characterization and engineering of a two-enzyme system for plastics depolymerization. Proc Natl Acad Sci U S A 117(41):25476–25485 32989159 10.1073/pnas.2006753117 PMC7568301

[63] Liu M, Yang S, Long L, Cao Y, Ding S (2018) Engineering a chimeric lipase-cutinase (Lip-Cut) for efficient enzymatic deinking of waste paper. BioResources 13(1):981–996

[64] Nath P, Dhillon A, Kumar K, Sharma K, Jamaldheen SB, Moholkar VS et al (2019) Development of bi-functional chimeric enzyme (CtGH1-L1-CtGH5-F194A) from endoglucanase (CtGH5) mutant F194A and Β-1,4-glucosidase (CtGH1) from *Clostridium thermocellum* with enhanced activity and structural integrity. Bioresour Technol 282(March):494–501 30897487 10.1016/j.biortech.2019.03.051

[65] Otsuka R, Imai S, Murata T, Nomura Y, Okamoto M, Tsumori H et al (2015) Application of chimeric glucanase comprising mutanase and dextranase for prevention of dental biofilm formation. Microbiol Immunol 59(1):28–36 25411090 10.1111/1348-0421.12214

[66] Baklouti Z, Delattre C, Pierre G, Gardarin C, Abdelkafi S, Michaud P et al (2020) Biochemical characterization of a bifunctional enzyme constructed by the fusion of a glucuronan lyase and a chitinase from *Trichoderma Sp*. Life 10(10):1–15 10.3390/life10100234 PMC7601620 33049934

[67] Bülow L, Ljungcrantz P, Mosbach K (1985) Preparation of a soluble bifuncrional enzyme by gene fusion. Bio/Technology 3(9):821–823

[68] Ho SN, Hunt HD, Horton RM, Pullen JK, Pease LR (1989) Site-directed mutagenesis by overlap extension using the polymerase chain reaction. Gene 77(1):51–59 2744487 10.1016/0378-1119(89)90358-2

[69] Engler C, Kandzia R, Marillonnet S (2008) A one pot, one step, precision cloning method with high throughput capability. PLoS ONE 3(11):e3647 18985154 10.1371/journal.pone.0003647 PMC2574415

[70] Kirchmaier S, Lust K, Wittbrodt J (2013) Golden GATEway cloning–a combinatorial approach to generate fusion and recombination constructs. PLoS ONE 8(10):e76117 24116091 10.1371/journal.pone.0076117 PMC3792108

[71] Joska TM, Mashruwala A, Boyd JM, Belden WJ (2014) A universal cloning method based on yeast homologous recombination that is simple, efficient, and versatile. J Microbiol Methods 100(1):46–51 24418681 10.1016/j.mimet.2013.11.013 PMC4521215

[72] Bryksin AV, Matsumura I (2010) Overlap extension PCR cloning: a simple and reliable way to create recombinant plasmids. Biotechniques 48(6):463–465 20569222 10.2144/000113418 PMC3121328

[73] Marquardt T, von der Heyde A, Elleuche S (2014) Design and establishment of a vector system that enables production of multifusion proteins and easy purification by a two-step affinity chromatography approach. J Microbiol Methods 105:47–50 25026273 10.1016/j.mimet.2014.07.008

[74] Chen X, Zaro JL, Shen WC (2013) Fusion protein linkers: property, design and functionality. Adv Drug Deliv Rev 65(10):1357–1369 23026637 10.1016/j.addr.2012.09.039 PMC3726540

[75] Xue Y, Peng J, Wang R, Song X (2009) Construction of the trifunctional enzyme associating the *Thermoanaerobacter ethanolicus* xylosidase-arabinosidase with the *Thermomyces lanuginosus* xylanase for degradation of arabinoxylan. Enzyme Microb Technol 45(1):22–27

[76] Wang R, Xue Y, Wu X, Song X, Peng J (2010) Enhancement of engineered trifunctional enzyme by optimizing linker peptides for degradation of agricultural by-products. Enzyme Microb Technol 47(5):194–199

[77] Lu P, Feng MG, Li WF, Hu CX (2006) Construction and characterization of a bifunctional fusion enzyme of Bacillus-sourced β-glucanase and xylanase expressed in *Escherichia coli*. FEMS Microbiol Lett 261(2):224–230 16907724 10.1111/j.1574-6968.2006.00367.x

[78] Lu P, Feng MG (2008) Bifunctional enhancement of a β-glucanase-xylanase fusion enzyme by optimization of peptide linkers. Appl Microbiol Biotechnol 79(4):579–587 18415095 10.1007/s00253-008-1468-4

